# REFLECTION AND INTENTION: END-OF-LIFE PLANNING IN FORMER CAREGIVERS OF PARENTS LIVING WITH DEMENTIA

**DOI:** 10.1093/geroni/igac059.840

**Published:** 2022-12-20

**Authors:** Emily Mroz, Tara Matta, Talha Ali, Amanda Piechota, Anissa Abboud, Shubam Sharma, Joan Monin, Terri Fried

**Affiliations:** Yale University, New Haven, Connecticut, United States; University of South Florida, Tampa, Florida, United States; Yale School of Medicine, New Haven, Connecticut, United States; Yale School of Public Health, New Haven, Connecticut, United States; Yale School of Public health, New Haven, Connecticut, United States; Kennesaw State University, Kennesaw, Georgia, United States; Yale School of Public Health, New Haven, Connecticut, United States; Yale School of Medicine, New Haven, Connecticut, United States

## Abstract

Family caregivers for parents living with Alzheimer’s Disease or Related Dementias (ADRD) provide support through health decline, care planning, and death. Guided by the Consensual Qualitative Research method and grounded in a life story framework, this study examines recalled caregiving experiences and descriptions of personal end-of-life planning in 32 midlife former caregivers of parents with ADRD (range: 40-65; 44% male). Former caregivers often expressed appreciation for end-of-life planning but varied in their engagement in planning since their loss. Descriptions of hesitation with planning were rooted in salient challenges from caregiving experiences and reflected relational concerns (e.g., about burdening others with care needs), planning complexity (e.g., perceiving too many factors to account for), or mortality denial (e.g., aversion to thinking more about death). These caregiving experiences may clarify the value of end-of-life planning while, in some cases, impeding decision-making, leading to gaps between former caregivers’ planning intentions and engagement.

